# Dose Calculation Accuracy of Beam Models in RadCalc for a 1.5 T MR-Linac

**DOI:** 10.3390/cancers16030526

**Published:** 2024-01-26

**Authors:** Jiwon Sung, Yeonho Choi, Jun Won Kim, Ho Lee

**Affiliations:** 1 Department of Radiation Oncology, Gangnam Severance Hospital, Yonsei University College of Medicine, Seoul 06273, Republic of Korea; pokemon30@yuhs.ac (J.S.); yunhoc12@yuhs.ac (Y.C.); junwon@yuhs.ac (J.W.K.); 2 Department of Radiation Oncology, Yonsei Cancer Center, Heavy Ion Therapy Research Institute, Yonsei University College of Medicine, Seoul 03722, Republic of Korea

**Keywords:** MR-linac, patient-specific quality assurance, online adaptive planning, independent dose verification software, beam model

## Abstract

**Simple Summary:**

The recent advancement to RadCalc version 7.1.4 marks a significant milestone as an independent dose verification tool tailored to encompass both mechanical and dosimetric intricacies of MR-linacs. Although previous assessments successfully validated the software’s efficacy on a 0.35 T MR-linac using v7.1.4, its validation for a 1.5 T MR-linac was restricted to v6.3. This discrepancy prompted an investigation into the software’s performance on a 1.5 T MR-linac due to the absence of a specific MR-linac beam-modeling protocol within RadCalc. This study’s novelty lies in the rigorous evaluation of calculation accuracy utilizing diverse beam-modeling techniques for the 1.5 T MR-linac. Results of this comprehensive analysis, showcasing RadCalc’s calculated doses and their alignment with expected measurements, robustly affirm its credibility and accuracy in dose calculations, particularly within the complex setting of the 1.5 T magnetic field.

**Abstract:**

The purpose of this study is to evaluate RadCalc, an independent dose verification software, for patient-specific quality assurance (PSQA) in online adaptive planning with a magnetic resonance linear accelerator (MR-linac) of a 1.5 T. Version 7.1.4 of RadCalc to introduce the capability to establish a beam model that incorporates MR field characteristics. A total of six models were established, with one using manufacturer-provided data and the others differing in percentage depth dose (PDD) data sources. Overall, two models utilized PDD data from the treatment planning system (TPS), and three used commissioned PDD data from gantry angles of 0° and 270°. Simple tests on a virtual water phantom assessed dose-calculation accuracy, revealing percentage differences ranging from −0.5% to −20.6%. Excluding models with significant differences, clinical tests on 575 adaptive plans (prostate, liver, and breast) showed percentage differences of −0.51%, 1.12%, and 4.10%, respectively. The doses calculated using RadCalc demonstrated similar trends to those of the PSQA-based measurements. The newly released version of RadCalc enables beam modeling that considers the characteristics of the 1.5 T magnetic field. The accuracy of the software in calculating doses at 1.5 T magnetic fields has been verified, thereby making it a reliable and effective tool for PSQA in adaptive plans.

## 1. Introduction

In recent years, Magnetic Resonance (MR)-guided radiotherapy (MRgRT) has gained increasing prominence within the clinical landscape, driven by the introduction of advanced systems such as the 0.35 T ViewRay MRIdian (Viewray Inc., Oakwood Village, OH, USA) and the 1.5 T Elekta Unity (Elekta AB, Stockholm, Sweden) [1,2,3,4]. This innovative modality leverages MR imaging to ascertain the patient’s precise anatomical positioning and the treatment target without subjecting the patient to additional imaging doses during the treatment. MR images afford superior soft tissue contrast relative to conventional kV/MV images employed in conventional radiotherapy; thus, enabling finer-grained tumor localization, even in proximity to soft tissue [5,6,7,8].

MRgRT also offers online adaptive radiotherapy (oART) based on daily MR images, which improves treatment accuracy by modifying the initial treatment plan according to the patient’s anatomical position on each treatment day [9,10,11]. To ensure the accuracy and safety of adaptive radiotherapy before delivering the treatment beam to the patient, two methods of patient-specific quality assurance (PSQA) are employed: measurement-based PSQA and calculation-based PSQA [12]. Since performing measurement-based PSQA in the treatment room while the patient is on the couch can be challenging, calculation-based PSQA is often the preferred method in oART. For performing calculation-based PSQA, various commercial software packages, known as secondary dose/MU calculation programs, are available. The American Association of Physicists in Medicine (AAPM) have also released reports in 2011 and 2019 that provide a review of various calculation algorithms used in these programs, guidelines on dose/MU verification, and recommendations on clinically reasonable action levels for calculation-based PSQA [13,14].

Recent studies have assessed the accuracy of dose calculations of secondary dose/MU calculation software in MRgRT. However, to the best of our knowledge, there is currently no commercial secondary dose calculation software that considers the MR effect in dose calculations. Although Hackett et al. [15], Pollitt et al. [16], and Yongbao Li et al. [17] used the collapsed cone (CC) algorithm in the Oncentra system, Mobius 3D, and RayStation to perform as the independent MU check for the Unity system, the accuracy of dose calculations is compromised as they all performed calculations without MR influence. In contrast, other studies incorporated MR influence into their dose calculations either by using Monte Carlo (MC) modeling capable of representing MR effects or by manual corrections. Wang et al. [18] and Friedel et al. [19] have developed a MC code with modeling of 0.35 T and 1.5 T magnetic field. Chen et al. [20] performed a secondary MU check by implementing a modified Clarkson integration algorithm to account for the magnetic field. Graves et al. [21] shifted the MLC and jaw positions of Elekta Unity to compensate for the influence on a 1.5 T magnetic field. Although these studies enhance the accuracy of dose calculations due to the slower calculation speed or the need for manual adjustments to consider the MR influence, the overall process of obtaining dose calculation results is time-consuming, limiting its applicability for fast online plan verification. In a recently published paper [22], researchers demonstrated its potential as a secondary dose verification software by improving computational speed and accuracy using a GPU-based in-house Monte Carlo engine. However, it is recognized that additional time is needed to achieve universal implementation in clinical settings.

Recently, RadCalc (LifeLine Software, Austin, TX, USA) released software version 7.1.4, which calculates dose monitor unit comparisons for MRgRT. This version incorporates magnetic field effects on the beam profiles by differentiating between crossline and inline profiles. These profiles exhibit different shapes due to the Lorentz force acting on the secondary scattered electrons. Previous studies utilized the previous version, v6.3, of the RadCalc software, which was unable to differentiate between inline and crossline profiles for beam modeling. It could not account for the cross-plane asymmetry induced by the 1.5 T magnetic field. This limitation led to significant discrepancies in the comparison of 2D dose maps with the MONACO treatment planning system (TPS) (Elekta AB, Stockholm, Sweden) [21]. In another study, version 7.1.4 of RadCalc was used for the dose calculation verification on the 0.35 T MRIdian ViewRay [23]. But, to the best of our knowledge, there is currently no secondary dose verification study available for this version of RadCalc on the 1.5 T Elekta Unity.

The Elekta Unity has a distinctive bore structure that sets it apart from conventional linacs. While the outer section adheres to a typical linac structure, the inner part integrates components such as the cryostat and various coils for MR image acquisition. Unity’s source-to-axis (SAD) is 143.5 cm, deviating from the standard linac’s 100 cm. Moreover, owing to a fixed patient couch and a bore size of 70 cm, the depth measured at a gantry of 0° during commissioning data acquisition is constrained to approximately 14.5 cm. Following Elekta’s guidelines, beam data are collected not only at 0° but also at 270°, adopting a comprehensive beam modeling approach distinct from conventional linacs. Currently, there is a lack of RadCalc beam modeling guidelines specifically tailored for Elekta Unity.

Therefore, this study aims to assess the accuracy of the dose calculations by creating various beam models that account for the influence of a 1.5 T MR field using RadCalc version 7.1.4. To achieve this, six beam models were established in RadCalc using different datasets, and their accuracy were evaluated through simple tests using a virtual water phantom. The calculated doses were compared to those obtained using MONACO TPS. Clinical tests were also conducted on 15 prostate, 14 liver, and 15 breast cancer patients, using the same methodology as the simple tests. Additionally, the correlation between the PSQA based on calculations and measurements was investigated.

## 2. Materials and Methods

### 2.1. Beam Modeling in RadCalc

A total of six Elekta Unity machines were modeled within RadCalc. Overall, five machines were configured using data from our Elekta Unity system, while the remaining one was created using pre-configured data, recommended by the manufacturer. To generate our Elekta Unity machine in RadCalc, we configured the overall machine-specific setting parameters using manufacturer-supplied data, but the parameters that differ from our system were adjusted to match our system. Specifically, since our Elekta Unity system was calibrated to 1 cGy/MU at SAD 143.5 cm with a depth of 5 cm using a 10 × 10 field size at a gantry of 0° and the calibration at the reference point is set to 0.87 cGy/MU with a reference depth of 10 cm. Additionally, various parameters, including D_max_ depth, jaw transmission, and MLC transmission, were also adjusted to match our system. A summary of these settings is provided in Table 1.

**Figure 1:**
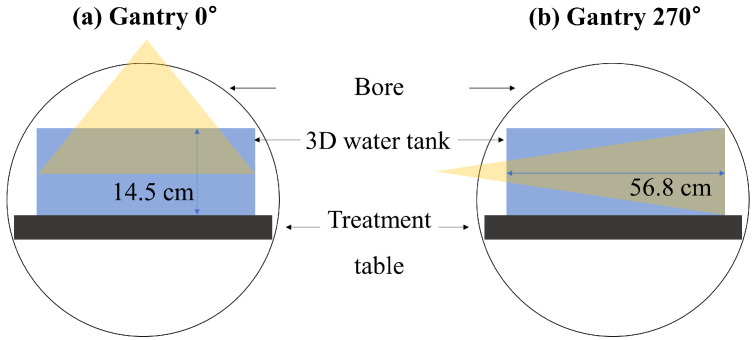
Limitation of the measurement depth at (**a**) a gantry of 0° and the measured field size at (**b**) a gantry of 270°.

After completing the machine configurations, beam modeling was performed for each machine using the collimator scatter factor (S_c_), phantom scatter factor (S_p_), off-axis ratio (OAR), and percentage depth doses (PDDs). As shown in Table 2, except for the PDDs, all of our five machines used the same beam data for beam modeling. As mentioned in the introduction section, due to the mechanical constraints of the Elekta Unity system and the limitations of MR-compatible QA tools, PDD measurements at a gantry of 0° are limited to a maximum depth of 14.5 cm (Figure 1a). Since the patient’s body depth often exceeds 14.5 cm, there are two methods to supplement these short PDD measurements. The first approach is to measure the PDDs at a gantry of 270°, the other approach is to use a virtual water phantom in the MONACO TPS (v5.51) to obtain deeper PDDs at a gantry of 0°. To determine which of these PDD datasets can generate the most optimal beam model in RadCalc, we utilized five different models: Models 1 and 2 were based on TPS data, Model 3 used PDD measurements obtained at a gantry of 270°, Model 4 relied on PDD measurements at a gantry of 0°, and Model 5 incorporated data from both a gantry at 0° and 270° angles for beam modeling.

To provide a more detailed explanation, the calculated PDD data were obtained using a virtual water phantom with dimensions of 60 × 30 × 23 cm^3^ for field sizes of 2 × 2, 3 × 3, 5 × 5, 10 × 10, 15 × 15, 22 × 22, 40 × 22, and 50 × 22 cm^2^, up to a depth of 45 cm at a gantry of 0°. These PDD data were post-processed using two methods: a smoothing tool and a fitting tool in OriginPro software v9.0 (OriginLab, Northampton, MA, USA). The post-processed PDD data were used to generate Models 1 and 2, respectively. All measurement data were commissioning data collected following the Elekta guidelines. The measured PDD data were obtained by scanning a 3D water phantom for field sizes of 2 × 2, 3 × 3, 5 × 5, 10 × 10, and 16 × 16 cm^2^ (As shown in Figure 1b, there is a limitation in the field size at 270°), up to a depth of 36 cm at a gantry of 270° and field sizes of 2 × 2, 3 × 3, 5 × 5, 10 × 10, 15 × 15, 22 × 22, 40 × 22, and 50 × 22 cm^2^, up to a depth of 13.5 cm at a gantry of 0°. Models 3 and 4 were established using the data measured at gantry angles of 270° and 0°, respectively. Model 5 was modeled using measured data at both 0° and 270°.

### 2.2. Evaluation of Beam Models for Virtual Phantom

The accuracy of the dose calculations using the beam models in RadCalc were evaluated by comparing them with the dose calculations in Elekta’s MONACO TPS. The virtual water phantom had dimensions of 60 × 30 × 23 cm^3^, and the treatment plans were configured with 3D CRT to deliver 100 MU per beam. At a gantry of 0°, eight beams were used, while five beams were employed at a gantry 270°. The SSD was 133.5 cm at a gantry of 0° and 113.1 cm at a gantry of 270°. The field size for each beam was 2 × 2, 3 × 3, 5 × 5, 10 × 10, 15 × 15, 22 × 22, 40 × 22, and 50 × 22 cm^2^ at a gantry of 0° and 2 × 2, 3 × 3, 5 × 5, 10 × 10, and 16 × 16 cm^2^ at a gantry of 270° in the TPS. The calculations were performed using a GPU-based Monte Carlo dose calculation algorithm (GPUMCD) [24,25,26] with a 0.3 cm grid size and 0.1% uncertainty.

**Figure 2:**
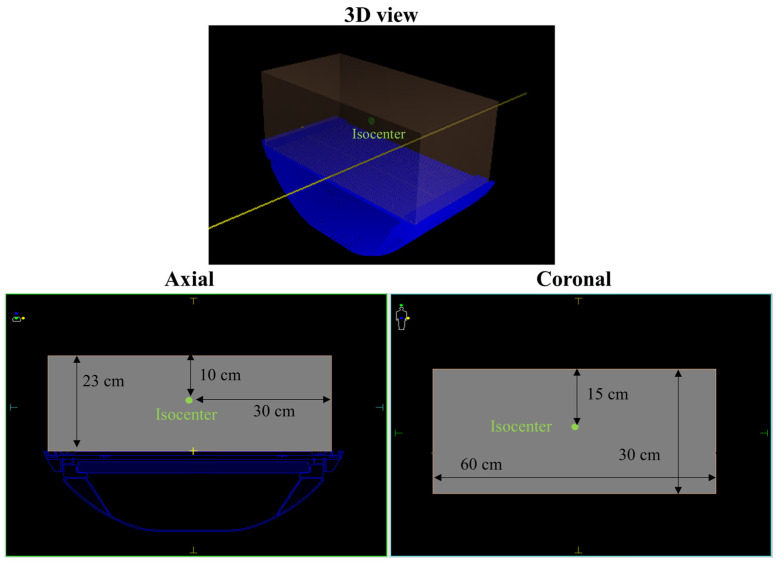
Sizes of the virtual phantom and position of the comparing point (=isocenter). Patient symbol in the upper left corner indicates the plane direction for the image (yellow dot: patient left direction, blue dot: patient anterior direction, and green dot: patient superior direction.

RadCalc performed dose calculations using the DICOM RT plan and RT structure files, which included all contoured structures, as well as the couch information, exported from the MONACO TPS. Each of the six beam models was calculated using the RadCalc dose calculation algorithm, a modified Clarkson integration (MCI) with an equivalent path [27,28]. Since RadCalc for Elekta Unity currently lacks a 2D gamma analysis tool, we conducted a point dose comparison. We assessed the percentage differences between the RadCalc and the TPS dose calculations at the isocenter, which was located 10 cm below the virtual phantom’s surface. Figure 2 shows the virtual phantom’s dimensions and the point of comparison within the virtual phantom. The percentage difference was calculated using the following formula:

$$\frac{\left(D_{RadCalc}-D_{TPS}\right)}{D_{TPS}}\times 100=Percentage\;difference\;\left(\%\right)$$

*D_TPS_* represents point dose calculated using the MONACO TPS based on the GPUMCD and *D_RadCalc_* represents the point dose calculated using RadCalc, based on the MCI.

### 2.3. Evaluation of Beam Models for Patients

**Figure 3:**
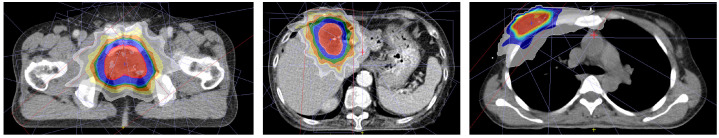
Target locations for prostate, liver, and breast treatment (blue cross mark in the target indicates the location of the comparison point).

To assess the accuracy of the calculated doses based on the beam models, a total of 575 daily adaptive plans were included in the evaluation. As shown in Table 3, these plans encompassed 15 prostate cancer cases, 14 liver cancer cases, and 15 breast cancer cases, all of which were treated using the Unity system. For the prostate cancer plans, a daily dose of 240 cGy was prescribed for the target in the prostate position, and 190 cGy for the target in the lymph node position or only 240 cGy for target, resulting in a total of 24 fractions. The liver cancer plans were designed with 10 fractions, each receiving a daily dose of 300–500 cGy, while the breast cancer plans consisted of 5 fractions with a daily dose of 200 cGy for boost treatment. The comparison point was positioned at the center of the target, which is illustrated in Figure 3. The percentage differences were calculated using the same formula as presented in Section 2.2.

### 2.4. Correlation with PSQA Based on Measurement

To investigate the correlation between the PSQA based on calculations and measurements, we compared the point doses measured using an ion chamber with those calculated using RadCalc for the 15 initial prostate treatment plans. The comparison point was the location with the highest dose within the target range.

In the MONACO TPS, a PSQA plan was generated based on the patient’s initial plan, and dose calculations were conducted. Subsequently, the DICOM RT plan and RT structure files of the plan were exported to RadCalc and calculated using Model 1, which provided the best match with the TPS calculations for both the virtual phantom and patient cases.

**Figure 4:**
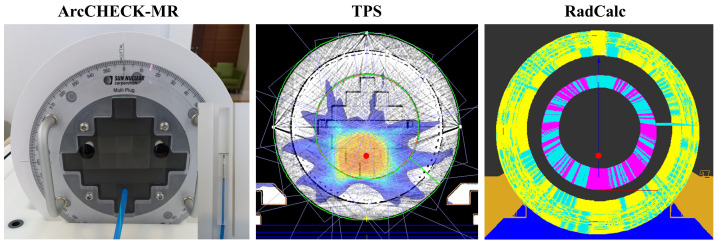
Setup and location for measurement using ArcCHECK-MR, as well as the corresponding TPS and RadCalc calculation location (red dot).

For measurement-based PSQA point doses were measured by inserting a Semiflex 3D (PTW 31021, PTW, Freiburg, Germany) chamber into the comparison position within the ArcCHECK-MR (Model 1220-MR, Sun Nuclear Corporation, Melbourne, FL, USA) with a MultiPlug, as shown in Figure 4.

## 3. Results

### 3.1. Evaluation of Beam Models in Virtual Phantom

Table 4 lists the percentage differences between the point doses calculated using each of the six models in RadCalc and those calculated using the TPS for each field size and gantry angle. The largest average difference was observed for Model 4, with −20.6% compared with the TPS, followed by Models 5 and 6, with differences of −13.0% and −9.2%, respectively. When compared by gantry angle, Model 4 had similar differences to the other models at a 0° gantry angle but exhibited a maximum difference of −73.7% and a minimum of −38.4% at a 270° gantry angle. Model 5 was similar to Model 4 at a 0° gantry angle but exhibited a difference of −44.9% at the maximum and −11% at the minimum gantry angle of 270 °. Model 6 exhibited the largest difference, with an average of −9.3% at a 0° gantry angle, and a difference similar to that at a 270° gantry angle with an average of −9.0%. In contrast, Models 1, 2, and 3 had lower differences, with average differences of −1.0%, −0.5%, and −1.0%, respectively. The maximum differences were −2.9%, −2.4%, and −2.6% for Models 1, 2, and 3, respectively.

### 3.2. Evaluation of Beam Models in Patients

**Figure 5:**
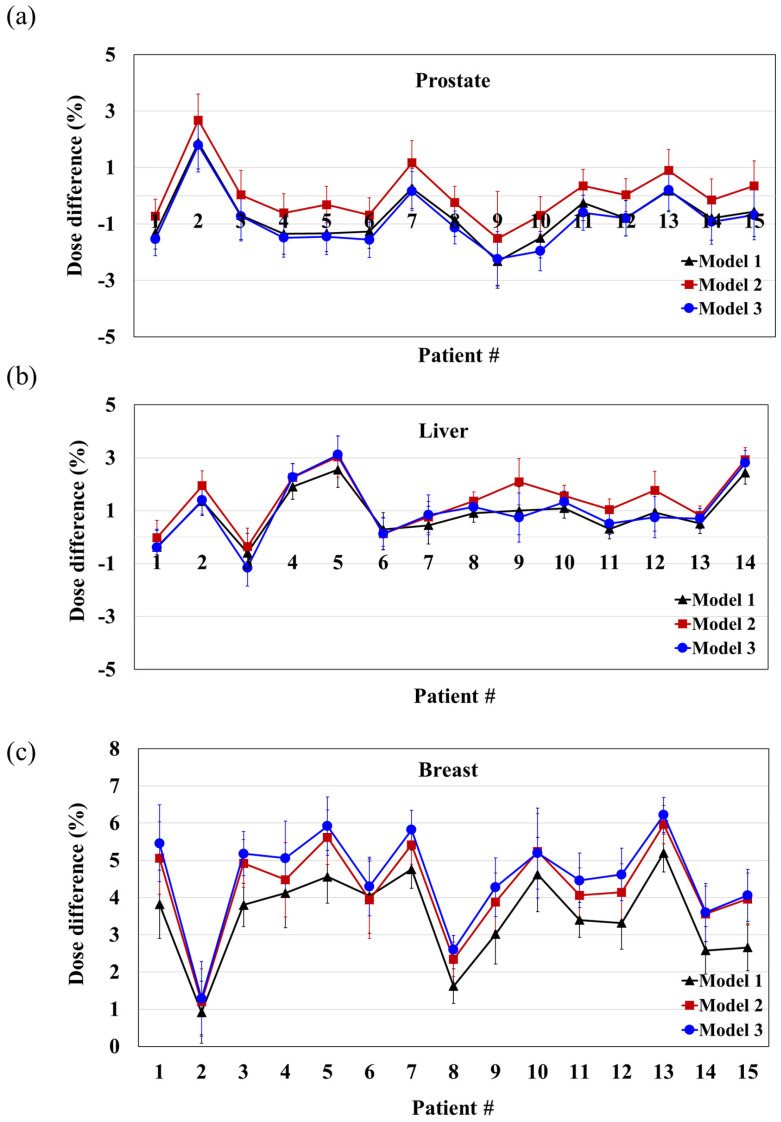
Differences between TPS and RadCalc for (**a**) prostate, (**b**) liver, and (**c**) breast cancer treatments for each patient. Each error bar represents the standard deviation. (#: patient number).

Based on the virtual phantom test, only Models 1, 2, and 3 were selected for further evaluation, because they showed an average difference of less than 5% from the TPS calculation. These models were then used to calculate the dose for the adaptive plans of patients with prostate, liver, and breast cancer, and the results were compared with the TPS calculation. As listed in Table 5, the difference between the three models at each site was approximately 1%, indicating similar results. The average differences in the three models for each organ were −0.51% for the prostate, 1.12% for the liver, and 4.10% for the breast, respectively, with the largest difference observed in the breast cancer case. Figure 5 demonstrates the difference between the TPS calculations for each patient and organ. The maximum absolute difference values for each model were 2.33%, 2.68%, and 2.24% for the prostate; 2.55%, 3.04%, and 3.12% for the liver; and 5.20%, 5.96%, and 6.22% for the breast. The results for the prostate and liver were within 5%, whereas those for the breast were within 7%.

### 3.3. Correlation with PSQA Based on Measurement

**Figure 6:**
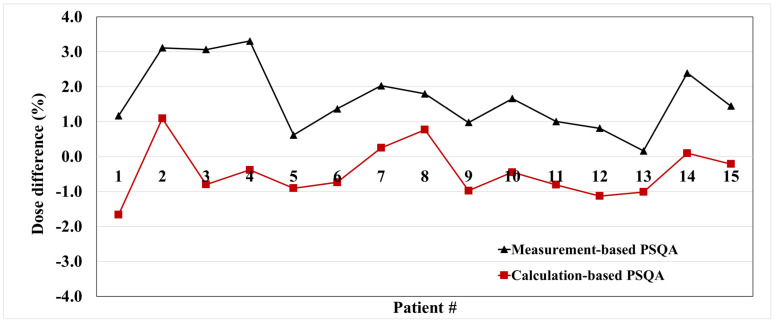
Correlation between measurement-based PSQA and calculation-based PSQA using Model 1. (#: patient number).

Figure 6 shows the trends in differences between measurement-based PSQA and TPS calculation, as well as between calculation-based PSQA and TPS calculation, for 15 initial prostate treatment plans. The measurement-based PSQA had an average difference of 1.66%, whereas the calculation-based PSQA using Model 1 had an average difference of −0.46%. The difference between measurement-based PSQA and calculation-based PSQA for a total of 15 plans generally exhibits a similar trend, with an average difference of around 2.11% ± 0.80%.

## 4. Discussion

Our study addresses three main points that distinguish it in the field. Firstly, to the best of our knowledge, RadCalc version 7.1.4 or above is the only commercial secondary dose/MU verification software capable of establishing beam models that consider the mechanical and dosimetric characteristics of a MR-linac. While it has been verified for 0.35 T MR-linac through a published study [23], it has not yet been validated for 1.5 T MR-linac. For the previous study on Elekta Unity, RadCalc v6.3 did not consider the effects of magnetic fields, resulting in a 67.3% gamma passing rate for 5%/5 mm criteria [21]. Therefore, we evaluated the accuracy of RadCalc version 7.1.4 through simple clinical tests on the 1.5 T MR-linac. Secondly, there are no commissioning protocols for MR-linacs in RadCalc. In the case of Elekta Unity, due to a fixed patient couch and a bore size of 70 cm, the depth measured at a gantry of 0° during commissioning, data acquisition are limited to around 14.5 cm. Consequently, following Elekta’s guidelines, beam data are gathered not only at 0° but also at 270° for a comprehensive beam modeling approach, which differs from conventional linacs. However, there are currently no RadCalc beam modeling guidelines available for Elekta Unity. Lastly, this is the first study to investigate the correlation between calculated doses from RadCalc and measured doses using an ion chamber, comparing calculation-based PSQA with measurement-based PSQA under identical conditions.

The accuracy of the dose calculations for the various beam models were evaluated on a virtual homogeneous phantom by comparing the doses obtained from RadCalc with those obtained from the TPS using different field sizes at gantry angles of 0° and 270°. The results showed that Models 4, 5, and 6 were less accurate than Models 1, 2, and 3. Model 4 was established using PDDs where the measurement depths were shortened to a gantry of 0° because of the limitations of the measurement equipment and table movement. Although Model 5 was built using measured PDDs of both a gantry of 0° and 270°, it did not reflect PDD data at deeper depth. Model 6 utilized configuration data provided by the manufacturer, which assumed a value of 0.79 cGy/MU at the reference depth. However, for our Unity machine, the actual value is 0.87 cGy/MU. Consequently, the doses calculated using our data were more consistent with those calculated using the manufacturer’s data.

Excluding Models 4, 5, and 6, which were modeled with insufficient or incorrect data, the accuracy of the dose calculations in a homogeneous phantom were evaluated for Models 1, 2, and 3. The results revealed that on average, Model 1 had a difference of −1.0 ± 0.9%, Model 2 had a difference of −0.5 ± 1.2%, and Model 3 had a difference of −1.0 ± 1.8%, compared to the TPS calculations. The Unity TPS used GPUMCD, which was accurate in dose calculations for 1.5 T MR, with differences within 1% for homogeneous phantoms and 2% for heterogeneous phantoms compared with GEANT4 [29]. Because the average differences between Models 1, 2, and 3, and the TPS calculations using GPUMCD were below 1%, this indicates that they accurately considered the MR effect in homogeneous materials. Also, we conducted a comparison between the calculated PDD data and the measured PDD data for four field sizes. A strong correspondence was observed in the overlapping regions (ranging from 0 cm to 13 cm). This high consistency in the overlapping regions suggests that the calculated PDD data can effectively substitute for the measured PDD data, particularly at depths where acquiring PDD measurements proves challenging. Furthermore, the calculation grid size can impact small fields (2 mm × 2 mm or 3 mm × 3 mm). Upon recalculation with a 1.5 mm × 1.5 mm grid size instead of a 3 mm × 3 mm grid size, the percentage difference showed a variation of approximately 0.3%.

To evaluate the accuracy of the dose calculation for inhomogeneous materials, we used 575 adaptive plans for 15 prostate, 14 liver, and 15 breast cancer patients. The average difference in dose calculations was significant for the breast plans at 4.10%, whereas the differences for the prostate and liver plans were −0.51% and 1.12%, respectively. In a 2017 study, the percentage differences in the calculated doses for lung and breast plans were 4.6% and 3.0%, respectively, which were higher than those in other regions [30]. These findings were consistent with our results. The TG 219 report revealed that dose calculation errors using the MCI algorithm, which is the RadCalc calculation algorithm, tended to be higher in heterogeneous regions and near the surface. This could explain the large differences observed in the breast treatment plans. The differences observed in the other two regions were also consistent with previously published results. The average difference between Models 1, 2, and 3 for each site was within 1%, indicating similar levels of calculation accuracy [31,32].

As reported in the previous study from 2019 [21], the average difference between RadCalc and MONACO TPS was 0.0% in Elekta Unity. This result was lower than our study’s average difference of 1.6%. The discrepancy in the number of plans contributed to these results, with our study involving 575 plans, while the 2019 study utilized 18 plans. Additionally, our study included 75 breast cases with an average error rate of 4.10%, slightly higher than other sites. This could be attributed to the target locations in the off-axis region and near the surface area, and the beam path passing through heterogeneous material. Moreover, the 2019 study employed RadCalc v6.3, which was limited by its inability to account for the MR influence on beam data, potentially resulting in reduced calculation reliability. Other recent studies [23] that utilized the same RadCalc version on the 0.35 T MR linac showed differences below 2% for homogeneous materials and below 5% for clinical cases excluding the lung. These results were similar to those in our study.

The study demonstrated that the new 7.1.4 version of RadCalc was more accurate than the previous version with a 1.5 T magnetic field. This is because the updated RadCalc version includes beam modeling that accurately reflects the beam characteristics in a 1.5 T MRI environment. However, further research is needed to investigate the region where the electron returns and electron streaming effects occur. These phenomena are caused by the magnetic field and significantly impact the boundary between different materials and the dose distribution around the jaw during breast treatments.

This study entailed a correlation analysis between point doses from measurement-based PSQA and calculation-based PSQA. The measurement-based PSQA and calculation-based PSQA demonstrated a consistent difference in each plan, indicating a similar trend. If the corresponding differences are corrected in the RadCalc software, it may be feasible to use RadCalc calculations as a substitute for measurements.

## 5. Conclusions

The newly released RadCalc software enables beam modeling that reflects the 1.5 T magnetic field and was confirmed to be a reliable and effective tool for PSQA in adaptive plans, owing to its ability to accurately calculate doses at 1.5 T magnetic fields.

## Figures and Tables

**Table 1:** Machine specific settings for our Elekta Unity system in RadCalc.

Parameter	Setting
Source axis distance	143.5 cm
Couch vertical zero position	0.0 cm
Volume average dose options	0.1 cm, automatically select best value
Clarkson radial sampling distance	0.1 cm
Clarkson angular sampling increment	5°
Clarkson radius used for primary dose	0.5 cm
Clarkson pixel size for intensity map	0.25 cm
Clarkson max angular step between control points	2.5°
Clarkson max leaf position change between control points	0.2 cm
Jaw transmission	0.006
MLC leaf transmission	0.006
Energy value (MV)	7 MV FFF
Reference SSD	133.5
D_max_ depth	1.4 cm
Reference depth	10 cm
Calibration @ reference	0.87 cGy/MU
Leaf width/position	0.717 cm width, range from −28.341 to +28.341
Allow fluence corrections for selected machine	Yes

**Table 2:** Data parameters used for each beam model in RadCalc (MD: measured data, CD: calculated data using the MONACO TPS, S_c_: collimator scatter factor, S_p_: phantom scatter factor, OAR: off-axis ratio, PDD: percentage depth dose).

Beam Modeling Parameters		Model					
		1	2	3	4	5	6
S_c_, S_p_, OAR		MD (same data except model 6)					Pre-configuration data
PDD	Data type	CD	CD	MD	MD	MD	
	Gantry angle	0°	0°	270°	0°	0° and 270°	
	Post-processed	Smoothing	Fitting	-	-	-	

**Table 3:** Patient information (*n*: number of cases).

Parameters		Prostate (*n* = 15)	Liver (*n* = 14)	Breast (*n* = 15)
Gender	Male	15 (100%)	9 (64%)	0 (0%)
	Female	0 (0%)	5 (36%)	15 (100%)
Age	30~39	0 (0%)	0 (0%)	3 (20%)
	40~49	0 (0%)	0 (0%)	3 (20%)
	50~59	2 (13%)	1 (7%)	5 (33%)
	60~69	5 (33%)	6 (43%)	4 (27%)
	70~79	8 (53%)	5 (36%)	0 (0%)
	80~89	0 (0%)	2 (14%)	0 (0%)
Stage	T0~Tis	0 (0%)	0 (0%)	5 (33%)
	T1~T2	6 (40%)	7 (50%)	10 (67%)
	T3~T4	9 (60%)	7 (50%)	0 (0%)
Target location	Right (Breast)	-	-	10 (67%)
	Left (Breast)	-	-	5 (33%)
Treatment technique	Palliative RT	0 (0%)	6 (43%)	0 (0%)
	Postoperative RT (boost)	0 (0%)	0 (0%)	15 (100%)
	Salvage RT	15 (100%)	8 (57%)	0 (0%)
Prescribed dose		Only 57.6 Gy or 57.6 Gy for target and 45.6 Gy for LN	30 Gy~50 Gy	10 Gy
Number of fractions		24	10	5
Total number of plans used in this study		360	140	75

**Table 4:** Percentage differences between point doses calculated using each of the six models in RadCalc and those calculated using the TPS, considering various field sizes and gantry angles. The reference point for comparison was positioned 10 cm below the virtual phantom’s surface, which corresponds to the isocenter location. (SD: standard deviation).

Gantry Angle	0°									270°					Mean	SD
Field Size (cm^2^)	2 × 2	3 × 3	5 × 5	10 × 10	15 × 15	20 × 20	22 × 22	40 × 22	50 × 22	2 × 2	3 × 3	5 × 5	10 × 10	16 × 16		
Model 1	−0.9	−2.4	−1.0	−0.8	−1.1	−1.0	−1.2	−0.8	−0.5	1.1	−2.9	−0.4	−0.8	−1.9	−1.0	0.9
Model 2	−0.9	−2.4	−1.0	−0.8	−1.1	−1.0	−1.3	−0.8	−0.5	2.0	−1.7	0.8	1.2	−0.1	−0.5	1.2
Model 3	−1.0	−2.6	−1.2	−0.9	−1.2	−1.1	−1.3	−0.9	−0.5	4.3	−0.9	−0.2	−1.9	−4.0	−1.0	1.8
Model 4	−0.9	−2.4	−1.0	−0.8	−1.1	−1.0	−1.3	−0.8	−0.5	−40.8	−38.4	−52.3	−72.8	−73.7	−20.6	28.7
Model 5	−0.9	−2.5	−1.1	−0.8	−1.1	−1.0	−1.3	−0.8	−0.5	−39.8	−11.0	−38.1	−44.9	−38.8	−13.0	18.2
Model 6	−10.3	−10.4	−9.2	−8.7	−9.2	−9.0	−9.2	−9.0	−8.7	−10.2	−10.1	−7.9	−7.8	−9.1	−9.2	0.8

**Table 5:** Differences between Models 1, 2, and 3 for prostate, liver, and breast cancer treatment plans (SD: standard deviation).

Site	Model	Average Dose Difference (%)	SD
Prostate	1	−0.71	1.0
	2	0.04	1.0
	3	−0.86	1.0
Liver	1	0.91	0.9
	2	1.43	1.0
	3	1.01	1.2
Breast	1	3.50	1.2
	2	4.25	1.3
	3	4.54	1.3

## Data Availability

All data generated or analyzed during this study are included in the article.
